# Characterization of a novel HIV-1 second-generation circulating recombinant form (CRF144_07C) in Ganzhou, China

**DOI:** 10.1016/j.gendis.2024.101443

**Published:** 2024-10-30

**Authors:** Xiaoyi Zhang, Hongmin Cao, Yating Chen, Chaoxian Lian, Ting Zeng, Junjie Liu, Junzhi Su, Qian Gao, Fengxiu Zhu, Yuning Zhang, Dandan Huang, Yanheng Zhou, Xin Chen

**Affiliations:** a Department of Epidemiology, School of Public Health and Health Management, Gannan Medical University, Ganzhou, Jiangxi 341000, China; b Department of Laboratory, Ganzhou Center for Disease Control and Prevention, Ganzhou, Jiangxi 341000, China; c Department of Pathogenic Biology, School of Basic Medical Sciences, Gannan Medical University, Ganzhou, Jiangxi 341000, China; d Shaanxi Engineering and Technological Research Center for Conversation and Utilization of Regional Biological Resources, School of Life Sciences, Yan'an University, Yan'an, Shaanxi 716000, China

Inter-subtype recombination is the main force for the complexity of HIV-1 genetic diversity, which increases the difficulty of preventing HIV-1 infection and administering antiretroviral therapy for people living with HIV. To date, 143 circulating recombinant forms (CRFs) have been reported globally, 43 of which were identified in China.^1^ Moreover, HIV-1 strains that are produced by second-generation combinations, including unique recombinant forms and CRFs, such as CRF105_0108, CRF123_0107, and CRF134_0185, have been commonly reported in recent years. The present study identified a novel second-generation recombinant form of HIV-1 comprising CRF07_BC and subtype C and designated CRF144_07C. To our knowledge, this was the first report of HIV CRFs comprising CRF07_BC and subtype C, further indicating the complexity of HIV genetic diversity in China.

Fourteen near full-length genomes were successfully amplified and sequenced from newly reported people living with HIV in Ganzhou, China, as described previously (Supplementary Materials and Methods).^2^ Among these 14 participants, most of them were male, ≥55 years, married, farmer, from Xinfeng county, infected through heterosexuals, and with viral load > 10^5^ copies/mL (Table S1). Maximum-likelihood tree using pure subtypes and CRFs comprising subtype B and subtype C as reference sequences showed that query sequences formed a unique cluster with a maximum parsimony bootstrap value of 100 %, located outside of the CRF07_BC cluster (Fig. 1). These results indicated that the query sequences might belong to a novel CRF divided from CRF07_BC and subtype C.

**Figure 1:**
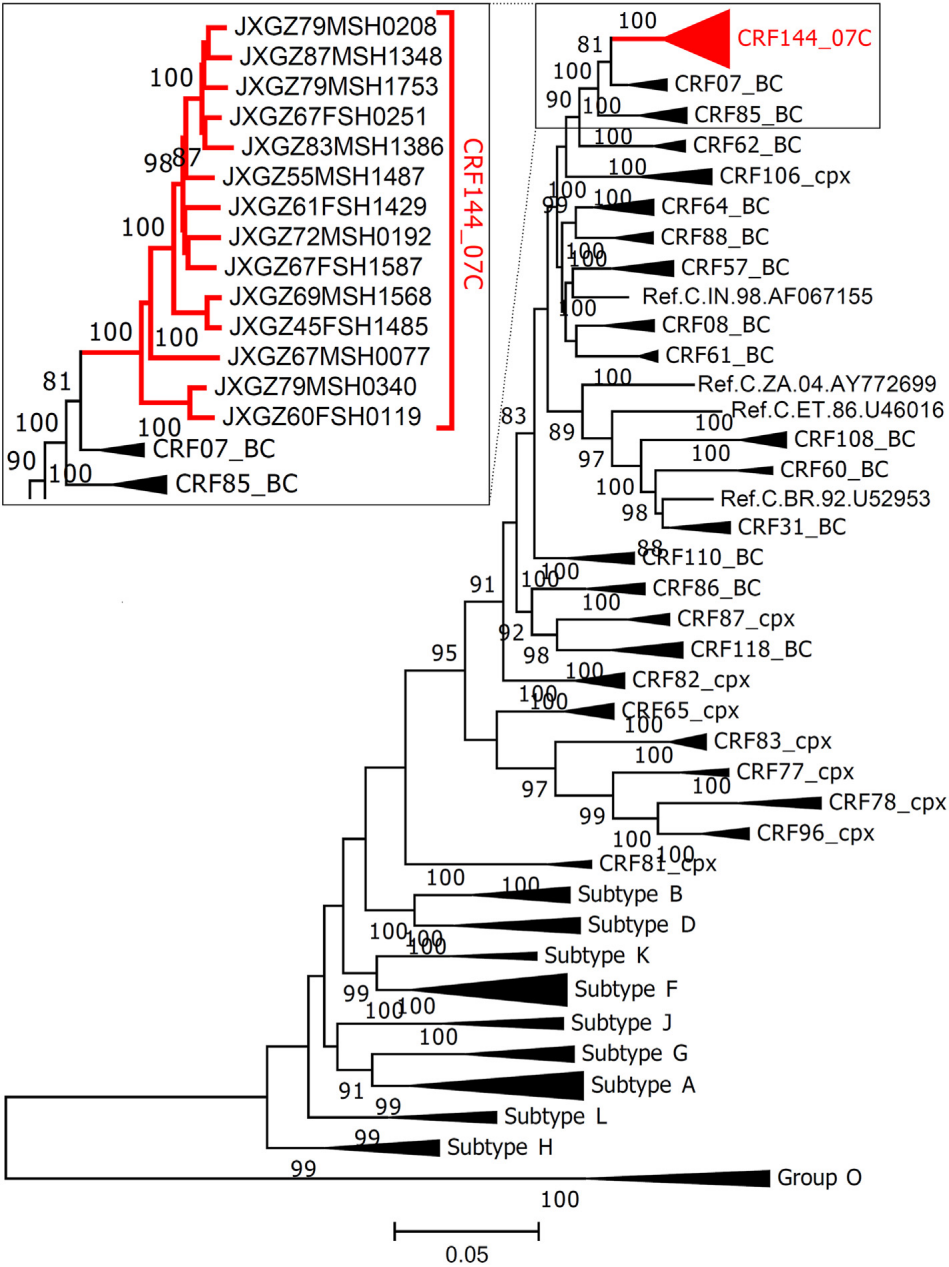
Maximum-likelihood trees based on HIV-1 near full-length genomes. The reference sequences included sequences of group O, subtypes A–L, and circulating recombinant forms (CRFs) comprising subtype B and subtype C. The reliability of the tree branches was evaluated by 1000 bootstrap replicates and only bootstrap values greater than 80 % were shown at each node. The branch of the potential novel CRF is marked in red.

Bootscan analyses revealed that these near full-length genomes comprised recombinants of three subtype B fragments and four subtype C fragments, with six identical breakpoints (Fig. S1A). Because they share the same mosaic structures, they could be designated as novel CRFs according to the HIV nomenclature proposal, which was named CRF144_07C. Compared with those of CRF07_BC, only two breakpoints of the mosaic structures of CRF144_07C were different (Fig. S1A), with the insertion of a subtype C fragment (nts 2188–2539 HXB2) into the backbone of CRF07_BC. Maximum-likelihood trees of the subregions of this novel CRF showed that subregion Ⅰ (nts 841–2187 HXB2) and Ⅲ (nts 2540–9024 HXB2) clustered with CRF07_BC, and subregion Ⅱ (nts 2188–2539 HXB2) clustered with subtype C (Fig. S2). In summary, CRF144_07C was a second-generation recombinant form of CRF07_BC and subtype C (Fig. S1B).

To estimate the emergence time of CRF144_07C, Bayesian evolutionary analyses were performed with the maximum subtype B fragment (nts 5817–6527 HXB2) and subtype C fragment (nts 3165–5816 HXB2). Maximum clade credibility trees revealed that the median time of the most recent common ancestor for the subtype B fragment (Fig. S3A) and subtype C fragment (Fig. S3B) were 2007.9 (95% highest probability density: 2003.8–2012.0) and 2007.5 (95% highest probability density: 2002.1–2011.8), respectively.

Although CRF144_07C was a newly identified HIV-1 strain, it seems to be prevalent in Ganzhou, China. According to the reanalysis of our previous study, the first patient infected with CRF144_07C was diagnosed in 2018 among outpatients in Ganzhou^3^ (Table S1), and the results of the present study showed that the median time of the most recent common ancestor for CRF144_07C was 2007 (Fig. S3), which indicates that CRF144_07C might have been prevalent in Ganzhou for approximately 15 years. Moreover, CRF144_07C was found to be prevalent in five different counties in Eastern, Western, and Southern Ganzhou, including Chongyi, Xinfeng, Ganxian, Ruijin, and Anyuan (Table S1). These findings showed that the newly identified CRF144_07C had become one of the main prevalent strains in Ganzhou, China.^4^

In the early stages of the pandemic, newly identified HIV-1 strains seemed to be highly relevant to the risk population. In the 1990s, HIV-1 CRF01_ AE and CRF07_ BC began to be prevalent in China, with the former occurring mainly among people who have heterosexual intercourse and the latter occurring mainly among people who inject drugs. Later, both strains spread to other risk populations and became the two main prevalent strains in China.^5^ Of the 15 individuals with CRF144_07C, 13 were aged 55 years and older (Table S1), and all of them had experienced heterosexual intercourse, suggesting that CRF144_07C might arise in the elderly population through heterosexual transmission. More extensive and systematic research was urgently needed to further understand whether CRF144_07C spread to other risk populations, as had been done for CRF01_AE and CRF07_BC.

In summary, we performed a molecular epidemiologic study of 17 newly reported people living with HIV in Ganzhou from 2020 to 2021. A novel second-generation recombinant form of HIV-1 comprising CRF07_BC and subtype C was identified and designated CRF144_07C. CRF144_07C might be one of the main prevalent strains in Ganzhou. Continuous and systematic research is urgently needed to understand the dynamics of CRF144_07C and to monitor the genetic diversity of HIV-1 in China.

## Ethics declaration

The protocol of this study was approved by the Ethics Committee of Gannan Medical University (2021320). Written informed consent for sample collection and subsequent analysis was obtained from all participants.

## Funding

This work was supported by the 10.13039/501100001809National Natural Science Foundation of China (No. 82060367), the Science and Technology Plan of Health Commission of Jiangxi Province, China (No. SKJP_220210624), and the Science and Technology Research Project of the Department of Education of Jiangxi Province, China (No. GJJ2201455, GJJ211527).

## CRediT authorship contribution statement

**Xiaoyi Zhang:** Investigation, Methodology, Writing – original draft. **Hongmin Cao:** Conceptualization, Writing – review & editing. **Yating Chen:** Investigation. **Chaoxian Lian:** Investigation. **Ting Zeng:** Investigation. **Junjie Liu:** Data curation, Resources. **Junzhi Su:** Data curation, Resources. **Qian Gao:** Data curation, Resources. **Fengxiu Zhu:** Data curation, Resources. **Yuning Zhang:** Methodology, Software, Funding acquisition. **Dandan Huang:** Funding acquisition, Methodology, Software. **Yanheng Zhou:** Funding acquisition, Methodology, Software. **Xin Chen:** Conceptualization, Writing – review & editing.

## Sequence data

The sequences amplified by this study were submitted to GenBank under accession nos. OR117365 to OR117378.

## Conflict of interests

The authors declared no conflict of interests.

## Figures and Tables

**Figure S1:**
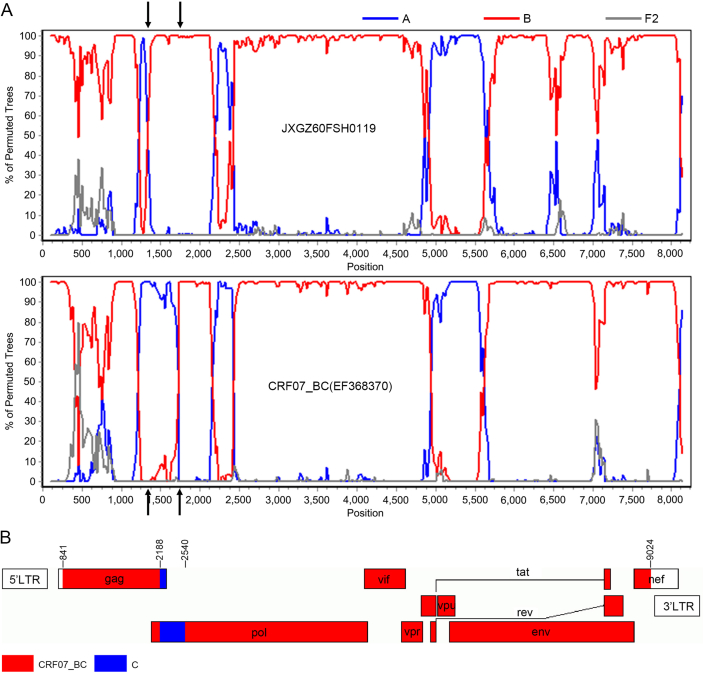
Recombinant structure of CRF144_07C. **(A)** Bootscan plots of representative sequences of CRF144_07C and CRF07_BC. Bootscan analysis was conducted using a window size of 200 bp and a step size of 20 bp along with the reference group of subtype B (GenBank accession nos. AB750367, AY180905, DQ007901, and DQ007902), subtype C (GenBank accession nos. KC898995, KF835515, U46016, and AF067155), and sub-subtype F2 (GenBank accession nos. AY371158, AJ249236, AJ249237, and AF377956). Distinct recombination breakpoints in the bootscan plots between CRF144_07C and CRF07_BC are marked with black arrows. **(B)** The mosaic map of CRF144_07C. The mosaic map was generated using the Recombinant HIV-1 Drawing Tool.

**Figure S2:**
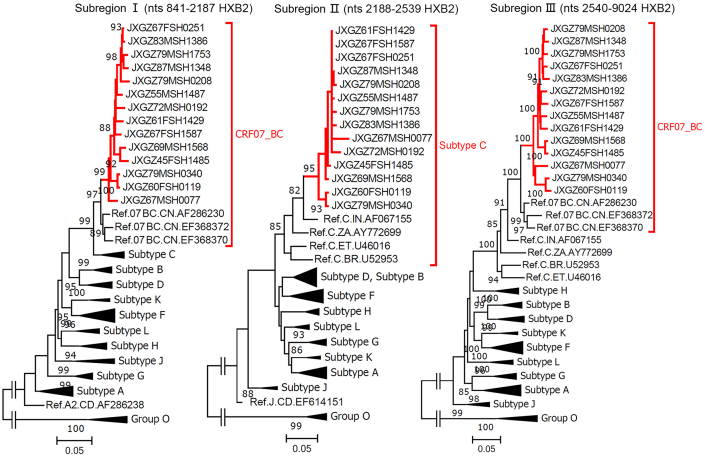
Maximum-likelihood trees based on subregions of CRF144_07C. The reference sequences included the sequences of group O, subtypes A–L, and CRF07_BC. The reliability of the tree branches was evaluated by 1000 bootstrap replicates, and only those with bootstrap values greater than 80 % are shown at each node. The CRF144_07C branch is marked in red.

**Figure S3:**
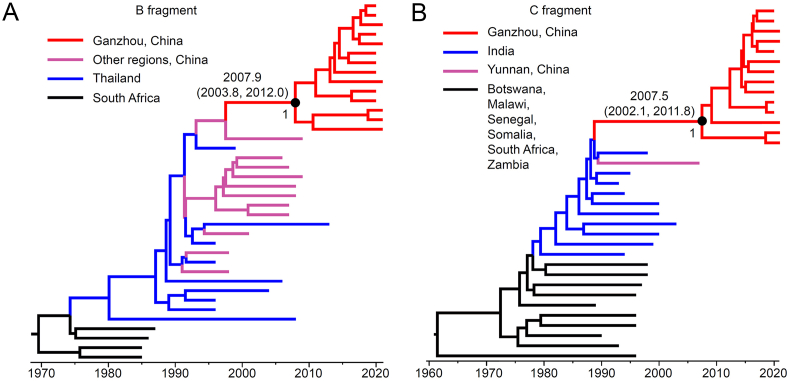
Maximum clade credibility trees based on fragments of CRF144_07C. Bayesian phylogeographic analysis was performed based on the longest fragments of subtype B origin (nts 5817–6527 HXB2) and subtype C origin (nts 3165–5816 HXB2). The timescale is shown at the bottom of the tree. The state posterior probability, the time of the most recent common ancestor, and the 95 % highest probability density for the nodes of CRF144_07C are indicated. The different colored lines indicate the sequences with different sample sites.
