# Supplementary material for: Characterization of a novel HIV-1 second-generation circulating recombinant form (CRF144_07C) in Ganzhou, China
Source: Genes Dis. 2024 Oct 30;12(4):101443. doi: 10.1016/j.gendis.2024.101443 (PMC11982966; doi:10.1016/j.gendis.2024.101443)
Supplement: Multimedia component 1 [file mmc1.docx]

**Supplementary Materials and Methods**

**Participants**

Blood samples from 529 newly reported people living with HIV were obtained from the Ganzhou Centre for Disease Control and Prevention in Ganzhou, China. The protocol of this study was approved by the Ethics Committee of Gannan Medical University (2021320). Written informed consent for sample collection and subsequent analysis was obtained from all participants.

Basic demographic data, including gender, age, marital status, occupation, education, residence and transmission route, were collected through questionnaires (Table S1), and five milliliters of peripheral blood was collected from each participant in EDTA2K plus vacuum tubes. The plasma samples were separated by centrifugation, sent to the laboratory through cold-chain transportation, and stored in an ultralow temperature refrigerator at -80°C until subsequent experiments.

**Amplification of the HIV-1** **near full-length genomes**

Viral RNA was extracted from the plasma samples using a High Pure Viral RNA Kit (Roche, Germany) and reverse transcribed into complementary DNA using a PrimeScriptTM Ⅱ 1st Strand cDNA Synthesis Kit (TaKaRa, Japan). The HIV-1 near full-length genomes was amplified by nested polymerase chain reaction using TransTaq® DNA Polymerase High Fidelity (TransGen, China), as described previously (PLoS One. 2008;3(1): e1420). Briefly, three overlapping genomic fragments were amplified independently, including the *gag-pol* fragment (nts 769–3338 HXB2), the *pol-vpu* fragment (nts 2483–6231 HXB2), and the *env-nef* fragment (nts 5861–9181 HXB2). The amplified products were tested with a 1% agarose gel. The possible positive samples were purified and sequenced by Tsing Ke Biotech Co., Ltd (Kunming, China).

The near full-length genomes were assembled by overlapping sequencing using the SeqMan software of the DNASTAR 7.1 package and were hand-curated to ensure the correct open reading frame according to the sequence chromatogram.

**Phylogenetic analysis**

Sequences of group O, subtypes A-L, and CRFs comprising subtype B and subtype C were downloaded from the HIV sequence database and used as reference sequences. After alignment, a maximum-likelihood tree was constructed with the Kimura two-parameter model using MEGA7.0. The reliability of the tree branches was evaluated by 1000 bootstrap replicates. For the potential novel CRFs and most similar sequences, bootscan analysis was performed based on 100 replicates with a 200 bp sliding window moving in steps of 20 nts using Simplot 3.5.1 to identify potential HIV-1 recombinants. Maximum-likelihood trees of subregions of the potential novel CRFs were constructed to confirm the subtype of the fragments that formed the CRFs. The mosaic maps of the potential novel CRFs were generated using the Recombinant HIV-1 Drawing Tool24.

**Phylogenetic analyses**

To estimate the most recent common ancestor (tMRCA) of the potential novel CRF, Bayesian phylogeographic analysis was performed based on the longest fragments of the pure subtypes using BEAST 1.7.525. The uncorrelated relaxed lognormal molecular clock was chosen, and each Markov chain Monte Carlo was run 200 million times, sampling every 20,000 generations. After all the programs were run, the posterior probability was calculated using the software Tracer v 1.4.1, and at the same time, the model was checked whether it had an effective sampling size. If the effective sampling size was greater than 200 or more, then the analysis results reached convergence, and the software Tree Annotator was used to summarize the maximum clade credibility (MCC) tree26. The MCC tree was viewed using FigTree 1.4.2 to view the origin of the sample and the location of recombination.
