# Supplementary material for: Characterization of a novel HIV-1 second-generation circulating recombinant form (CRF144_07C) in Ganzhou, China
Source: Genes Dis. 2024 Oct 30;12(4):101443. doi: 10.1016/j.gendis.2024.101443 (PMC11982966; doi:10.1016/j.gendis.2024.101443)
Supplement: Multimedia component 2 [file mmc2.docx]

**Table S1. The demographic information of patients infected with CRF144_07C in Ganzhou, China.**

| **Patient number** | **Sampling year** | **Sampling county** | **Age (years)** | **Gender** | **Occupation** | **Marital status** | **Risk factor** | **Viral load (copy/mL)** | **GenBank accession nos.** |
| --- | --- | --- | --- | --- | --- | --- | --- | --- | --- |
| JXGZ67FSH0251 | 2021 | Chongyi | 67 | Female | Farmer | Married | Heterosexual | 632000 | OR117365 |
| JXGZ79MSH0340 | 2021 | Xinfeng | 79 | Male | Farmer | Married | Heterosexual | 111000 | OR117366 |
| JXGZ79MSH0208 | 2021 | Xinfeng | 79 | Male | Farmer | Married | Heterosexual | 179000 | OR117367 |
| JXGZ72MSH0192 | 2021 | Xinfeng | 72 | Male | Farmer | Married | Heterosexual | 86200 | OR117368 |
| JXGZ79MSH1753 | 2020 | Xinfeng | 79 | Male | Teacher | Married | Heterosexual | >10000000 | OR117369 |
| JXGZ67MSH0077 | 2021 | Xinfeng | 67 | Male | Teacher | Married | Heterosexual | 147000 | OR117370 |
| JXGZ60FSH0119 | 2021 | Ganxian | 60 | Female | Farmer | Married | Heterosexual | 157000 | OR117371 |
| JXGZ67FSH1587 | 2020 | Xinfeng | 67 | Female | Farmer | Married | Heterosexual | 214000 | OR117372 |
| JXGZ69MSH1568 | 2020 | Xinfeng | 69 | Male | Farmer | Married | Heterosexual | 15100 | OR117373 |
| JXGZ55MSH1487 | 2020 | Xinfeng | 55 | Male | Worker | Unmarried | Heterosexual | 223000 | OR117374 |
| JXGZ45FSH1485 | 2020 | Ruijin | 45 | Female | Businessman | Married | Heterosexual | 211000 | OR117375 |
| JXGZ61FSH1429 | 2020 | Xinfeng | 61 | Female | Farmer | Married | Heterosexual | 126000 | OR117376 |
| JXGZ83MSH1386 | 2020 | Xinfeng | 83 | Male | Farmer | Married | Heterosexual | 54300 | OR117377 |
| JXGZ87MSH1348 | 2020 | Xinfeng | 87 | Male | Farmer | Married | Heterosexual | 4330 | OR117378 |
| JXGZ40MSH8 ^a^ | 2018 | Anyuan | 40 | Male | Farmer | Married | Heterosexual | 26000 | MT611507 |

^a^ Results from our previous study (AIDS Research and Human Retroviruses, 2021, 37(2): 147-50).
